# The Effects of the KCNQ Openers Retigabine and Flupirtine on Myotonia in Mammalian Skeletal Muscle Induced by a Chloride Channel Blocker

**DOI:** 10.1155/2012/803082

**Published:** 2012-03-25

**Authors:** Tzu-Rong Su, Wen-Shan Zei, Ching-Chyuan Su, George Hsiao, Min-Jon Lin

**Affiliations:** ^1^Tian-Sheng Memorial Hospital, Tong-Kang, Pintong, Taiwan; ^2^School of Biomedical Sciences, Chung Shan Medical University, Taichung 402, Taiwan; ^3^Department and Graduate Institute of Pharmacology, College of Medicine, Taipei Medical University, Taipei, Taiwan; ^4^Department of Medical Research, Chung Shan Medical University Hospital, Taichung, Taiwan

## Abstract

The purpose of this study was to investigate the effect of KCNQ (potassium channel, voltage-gated, KQT-like subfamily) openers in preventing myotonia caused by anthracene-9-carboxylic acid (9-AC, a chloride channel blocker). An animal model of myotonia can be elicited in murine skeletal muscle by 9-AC treatment. KCNQ openers, such as retigabine and flupirtine, can inhibit the increased twitch amplitude (0.1 Hz stimulation) and reduce the tetanic fade (20 Hz stimulations) observed in the presence of 9-AC. Furthermore, the prolonged twitch duration of skeletal muscle was also inhibited by retigabine or flupirtine. Lamotrigine (an anticonvulsant drug) has a lesser effect on the muscle twitch amplitude, tetanic fade, and prolonged twitch duration as compared with KCNQ openers. In experiments using intracellular recordings, retigabine and flupirtine clearly reduced the firing frequencies of repetitive action potentials induced by 9-AC. These data suggested that KCNQ openers prevent the myotonia induced by 9-AC, at least partly through enhancing potassium conductance in skeletal muscle. Taken together, these results indicate that KCNQ openers are potential alternative therapeutic agents for the treatment of myotonia.

## 1. Introduction

KCNQ (potassium channel, voltage-gated, KQT-like subfamily) potassium channels (Kv7) play a major role in controlling membrane excitability and thus represent interesting drug targets for the treatment of epilepsy and neuropathic pain. [1–3]. To date, in mammals, five members of the KCNQ family have been reported [1]: Kv7.1 to Kv7.5. These are potassium (K^+^) channel proteins that are widely distributed in the brain, inner ear, heart, pancreas, lung, and placenta [4–8]. Retigabine and flupirtine, voltage-dependent KCNQ K^+^ channel (Kv.7) openers, exert anticonvulsant and analgesic actions in the central nervous system [9–11]. Retigabine also has antinociceptive effects in rat models of persistent and chronic pain. The antinociceptive effects associated with retigabine administration were reversed by coadministration of KCNQ blockers [10–13]. Flupirtine, a compound that is structurally similar to retigabine, enhances the activation of KCNQ K^+^ channels. This drug can prevent and suppress seizures in both the kainic acid and flurothyl models of symptomatic neonatal seizures [14] and is a centrally-acting, nonopioid analgesic that may prove useful in the treatment of a variety of pain states [15]. In addition to anticonvulsant and analgesic effects, several studies have shown that retigabine and flupirtine exert neuroprotective effects on central neurons [16–20]. Recent studies have reported that Kv7.1 [21] and Kv7.5 [22, 23] transcripts are expressed in adult skeletal muscle. Transcript levels of Kv7.1 and Kv7.5 are increased during *in vitro* proliferation and differentiation of rat myoblasts [24]. More recently, Iannotti et al. [25] showed that Kv7 K^+^ channels are expressed in differentiating C_2_C_12_ cells and myotubes [25]. However, the functional role of KCNQ K^+^ channels in mammalian skeletal muscle remains unknown. K^+^ channels may play a role in the patterns of muscle contraction and relaxation and potentially modulate the resting membrane potential of skeletal muscle cells [26]. In addition to K^+^ channels, chloride (Cl^−^) conductance accounts for more than 70% of the resting conductance in mammalian skeletal muscle. Myotonia occurs naturally in several species (humans, goats, mice) as a result of a genetic deficiency in the skeletal muscle CLC-1 Cl^−^ channel, a disease termed myotonia congenita in humans [27–30]. An animal model of myotonia in mammalian skeletal muscle can be induced by treatment with the Cl^−^ channel blocker 9-anthracene carboxylic acid. In the present studies, the effects of KCNQ K^+^ openers on Cl^−^ channel blocker-induced myotonia were investigated. It was found that KCNQ K^+^ openers but not lamotrigine (an anticonvulsant drug with no effect on KCNQ channel activity) inhibited muscle myotonia and the firing frequency of repetitive action potentials induced by the Cl^−^ channel blocker.

## 2. Materials and Methods

### 2.1. Mouse Phrenic Nerve-Diaphragm Preparations

ICR strain mice (17–22 g) were sacrificed inhaled carbon dioxide. Phrenic nerve-diaphragm preparations were isolated and suspended in an organ bath containing 10 mL modified Krebs' solution at 36 ± 1°C and oxygenated with carbogen (95% O_2_ + 5% CO_2_). The composition of the modified Krebs' solution was the following (in mM): NaCl (131), KCl (4.8), MgSO_4_ (1.2), CaCl_2_ (1), NaHCO_3_ (12.5), and glucose [11], with pH of 7.40 ± 0.1 when oxygenated. The isolated diaphragm was initially rinsed 3-4 times with the modified Krebs solution. Each isolated diaphragm was stretched to optimal length by applying a preload force of 2 g, and the peak isometric twitch tension was measured during indirect (phrenic nerve) or direct (muscle) stimulation. Stimulation was performed with a supramaximal constant-voltage pulse (a duration of 0.02 ms for indirect and 0.5 ms for direct stimulation) using a Grass S88 stimulator. Tetanic muscle contraction was achieved by using supramaximal stimulation at 20 Hz with a 3 s train duration. The phrenic nerve was stimulated electrically through a circular bipolar platinum electrode when indirect twitch tension was elicited. A pair of linear bipolar platinum stimulating electrodes was used when direct stimulation was performed. When direct stimulation was performed, the diaphragm was pretreated with 50 *μ*M d-TC to block the effect of the nerve and the influence of nerve stimulation. Contractions were recorded isometrically with a Grass force-displacement transducer (FT. 03C) on a Gould TA240 polygraph. The tetanic contraction maintenance (TCM) index was calculated as the last peak tension/initial peak tension. TD_50_ (twitch duration 50%) was calculated as the twitch duration at 50% of the amplitude. The stability of the twitch tension for at least 20–30 min was confirmed before further experimental procedures.

### 2.2. Action Potentials Recordings

Isolated diaphragms were pinned out on a Sylgard (Dow Corning Corporation, USA) plate at the bottom of a recording chamber, which contained oxygenated modified Krebs solution. Transmembrane action potentials were measured by an intracellular glass microelectrode with a high impedance amplifier (Axoclamp 2B, Axon Instruments, USA) in bridge mode. Borosilicate microelectrodes (GC150, Warner, USA) were fabricated using a Sutter P87 electrode puller (Sutter Instruments, USA). Glass microelectrodes were filled with 3 mol/L KCl and had impedances between 10–15 MΩ. Single or multiple action potentials were evoked by intracellular injection of depolarising current pulses (20 nA; 200 ms). The signals of membrane potentials and action potentials were digitised at 20–100 kHz and stored using DigiData 1322 A (Axon Instruments). Data were analysed using the pClamp 9.0 software (Axon Instruments).

### 2.3. Drugs and Chemicals

Retigabine was purchased from LKT laboratories (USA). Flupirtine, and lamotrigine were purchased from Tocris Bioscience, Inc. (UK). D-tubocurarine was purchased from Fluka Inc. (USA). Retigabine, Flupirtine and lamotrigine were dissolved in dimethyl sulfoxide (DMSO); d-tubocurarine was dissolved in distilled water.

### 2.4. Statistics

The data are provided as the mean ± S.E.M. Statistically significant of differences were evaluated using a paired or unpaired Student's *t*-test. When more than one group was compared with one control, significance was evaluated using one-way analysis of variance (ANOVA). Probability values (*P*) less than 0.01 were considered significant.

## 3. Results

### 3.1. Effects of KCNQ Openers on the Muscle Contractions Induced by Anthracene-9-Carboxylic Acid (9-AC)

**Figure 1:**
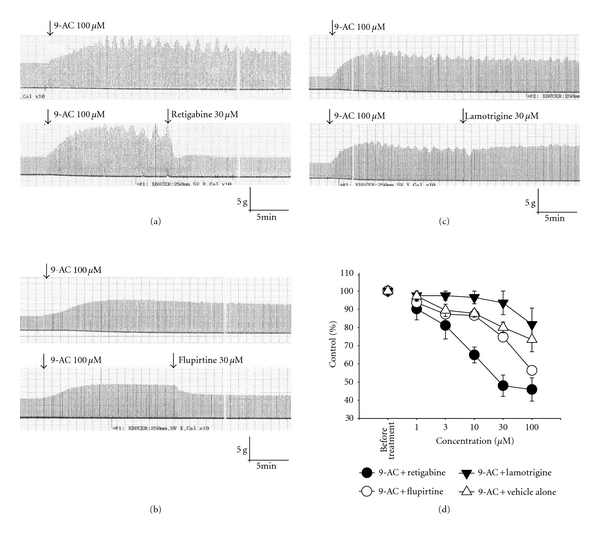
Effects of retigabine and flupirtine on the anthracene-9-carboxylic-acid (9-AC)-induced myotonia. Representative muscle contraction traces recorded from mouse phrenic nerve-diaphragm preparations are shown in (a)–(c). The addition of 9-AC (0.1 mM) increased the amplitude of muscle contractions. Treatment with retigabine (0.03 mM, (a)) and flupirtine (0.03 mM, (b)) resulted in an inhibition of the increased amplitude of muscle contractions induced by 9-AC. Lamotrigine (0.03 mM) has no inhibitory effect on the muscle amplitude induced by 9-AC (c). (d) Dose-response effects of retigabine, flupirtine, and lamotrigine on 9-AC-induced myotonia. Percent of control (*y*-axis): the twitch amplitude ratio of after treatment (retigabine, flupirtine, or lamotrigine) to before treatment (9-AC alone).

**Figure 2:**
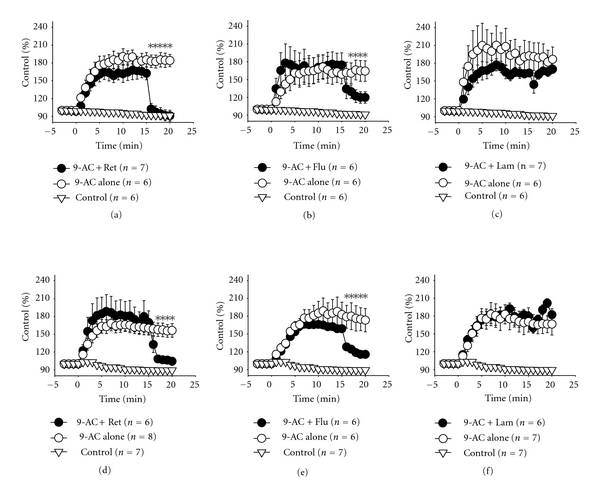
The effects of retigabine, flupirtine and lamotrigine on the statistical time-course curves of indirect-(nerve) and direct-(muscle) stimulated muscle contractions induced by 9-AC (0.01 mM). (a)–(c) and (d)–(f) are the muscle tensions elicited by indirect and direct stimulation at 0.1 Hz, respectively. Retigabine (0.03 mM, (a, d)) and flupirtine (0.03 mM, (b, e)), but not lamotrigine (0.03 mM, (c, f)), significantly inhibited the 9-AC-induced increase in muscle amplitude. 9-AC was applied at time zero. Retigabine, flupirtine, or lamotrigine was applied at time 15 min. *P < 0.01 as compared with 9-AC treatment alone. Control: no chemical treatment.

**Figure 3:**
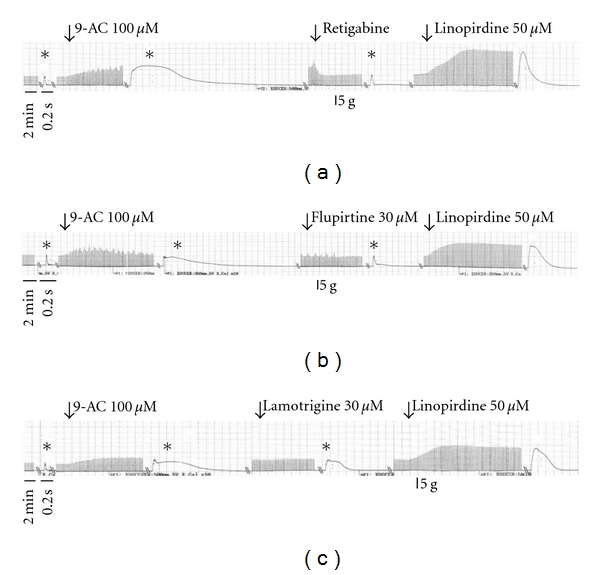
The effects of retigabine, flupirtine, and lamotrigine on the duration of isometric twitches induced by 9-AC (0.1 mM). The representative traces of muscle twitches showed the effects of retigabine (0.03 mM, (a)), flupirtine (0.03 mM, (b)), and lamotrigine (0.03 mM, (c)) on the prolonged duration of muscle twitches induced by 9-AC. Retigabine and flupirtine shortened the duration of muscle twitches in the presence of 9-AC. Linopirdine (0.05 mM), a KCNQ blocker, antagonises the effects of retigabine and flupirtine on the duration of muscle twitches. *a high-resolution time scale: 200 msec.

**Figure 4:**
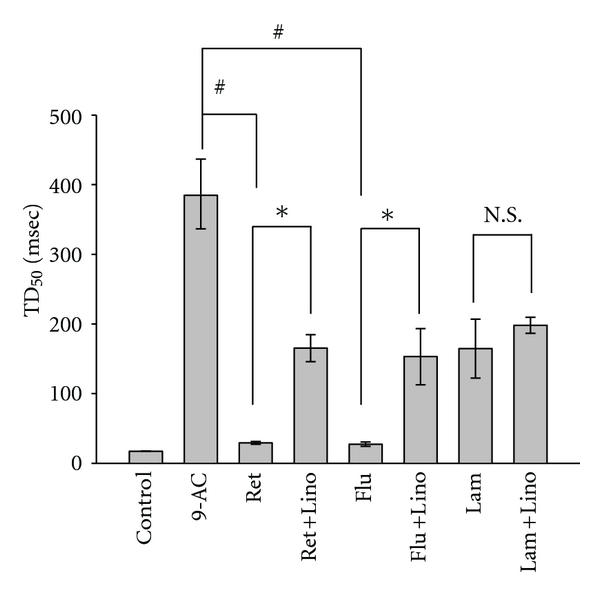
Statistical bar graph of the effects of KCNQ openers, lamotrigine and linopirdine (0.05 mM) on the twitch duration (TD_50_) of the muscle induced by 9-AC (0.1 mM). Control: no chemical treatment; 9-AC: 9-AC treatment alone; Ret: retigabine (0.03 mM); Flu: flupirtine (0.03 mM); Flu-lino: flupirtine plus linopirdine; Lam: lamotrigine; Lam-lino: lamotrigine plus linopirdine. TD_50_: twitch duration at 50% of the amplitude. ^#^ *P* < 0.05 as compared with 9-AC alone; *P < 0.01 as compared with retigabine (ret) or flupirtine (flu); N.S: no significance.

9-AC, a potent myotonia inducer, can cause increased membrane exicabibity and slowed muscle relaxation. The application of 9-AC (0.1 mM) caused an increase in twitch amplitude under direct stimulation conditions at a frequency of 0.1 Hz (Figure 1). Treatment with retigabine (Ret; Figure 1(a)), and Flupirtine (Flu; Figure 1(b)) but not lamotrigine (Lam; Figure 1(c)) significantly inhibited the increased amplitude of muscle contraction induced by 9-AC. Figure 1(d) shows the dose-response relationship of KCNQ openers and lamotrigine.  Figure 2 shows the time course of muscle force responses to indirect (Figures 2(a)–2(c)) and direct (Figures 2(d)–2(f)) stimulation. The effects of retigabine and Flupirtine on the response of 9-AC-treated muscle are similar in both indirect (*via* nerve) and direct (*via* muscle) stimulation. The maximal twitch amplitudes induced by 9-AC from three independent experiments groups were 188.3 ± 28.4, 166.2 ± 6.0, and 192.4 ± 9.6% of the control. Treatment with the KCNQ openers retigabine (Figures 2(a) and 2(d)) and flupirtine (Figures 2(b) and 2(e)), but not the anticonvulsant drug lamotrigine (182.4 ± 10.1%; Figures 1(c) and 2(f)), significantly inhibited the twitch amplitude induced by 9-AC (indirect stimulation: from 167.7 ± 15.8 to 91.1 ± 9.2% for Ret and 176.1 ± 20.7 to 119.9 ± 9.7% for Flu; direct stimulation: from 188.3 ± 28.4 to 104.4 ± 6.9% for Ret and 166.2 ± 6.0 to 115.65 ± 3.5% for Flu). The prolongation of the relaxation period in skeletal muscle induced by 9-AC (TD_50_: 386.6 ± 50.1 ms) is also significantly inhibited by treatment with retigabine (31.3 ± 1.9 ms) and flupirtine (29.3 ± 3.2 ms) (Figures 3 and 4). Linopirdine (Lino), a KCNQ blocker, reversed the effects of retigabine and flupirtine on the twitch duration (TD_50_) induced by 9-AC (Ret + Lino: 167.1 ± 19.5 ms; Flu + Lino: 155.0 ± 40.3 ms). Lamotrigine inhibits the prolonged relaxation period induced by 9-AC to a lesser extent than retigabine or flupirtine do (Figure 4). However, linopirdine cannot reverse the effects of lamotrigine on the inhibition of twitch duration induced by 9-AC (Figure 4).

### 3.2. Effects of KCNQ Openers on the Muscle Contractions Induced by 9-AC at 20 Hz Stimulation

**Figure 5:**
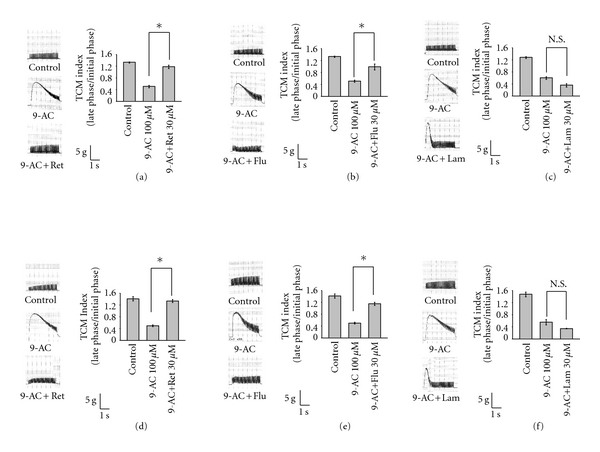
The effects of retigabine, flupirtine and lamotrigine on repetitive muscle contractions evoked by indirect (nerve)- and direct (muscle)- stimulation at a frequency of 20 Hz in the presence of 9-AC (0.1 mM). (a)–(c) and (d)–(f) are the muscle contractions evoked by the indirect and direct stimulation at a frequency of 20 Hz with 3 sec duration, respectively. (a, d): retigabine (0.03 mM); (b, e): flupirtine (0.03 mM); (c, f): lamotrigine (0.03 mM). TCM index: tetanic contraction maintenance index. *P < 0.01 as compared with 9-AC alone; N.S: no significance.

In the skeletal muscle without any chemical treatment, the typical tetanic contraction maintenance (TCM) index by direct and indirect stimulation at 20 Hz ranged from 1.28–1.47 (1.31 ± 0.03 for indirect stimulation, *n* = 18; 1.43 ± 0.08 for direct stimulation; Figures 5(a)–5(f)). The application of 9-AC caused a fade of tetanic contractions (muscle unable to sustain a contraction continuously) both in response to indirect (Figures 5(a)–5(c)) and direct (Figures 5(d)–5(f)) stimulation at 20 Hz (TCM index: 0.53 ± 0.04 for indirect stimulation, *n* = 21; 0.51 ± 0.05 for indirect stimulation, *n* = 21). Treatment with retigabine and flupirtine, but not lamotrigine, can reverse the fade of tetanic contractions induced by 9-AC. The TCM index values of 9-AC-treated diaphragm increase from 0.50 ± 0.04 to 1.34 ± 0.09 for indirect stimulation and 0.49 ± 0.03 to 1.33 ± 0.05 for direct stimulation after retigabine treatment; additionally, flupirtine treatment increases TCM index values from 0.50 ± 0.03 to 0.99 ± 0.09 for indirect stimulation and 0.50 ± 0.03 to 1.14 ± 0.05 for direct stimulation (Figure 5). Treatment with lamotrigine cannot reverse the TCM index values of 9-AC-treated diaphragms, which were observed as 0.60 ± 0.04 to 0.36 ± 0.05 for indirect stimulation and 0.55 ± 0.09 to 0.34 ± 0.04 for direct stimulation.

### 3.3. Effects of KCNQ Openers on the Frequency of Action Potentials Induced by 9-AC

**Figure 6:**
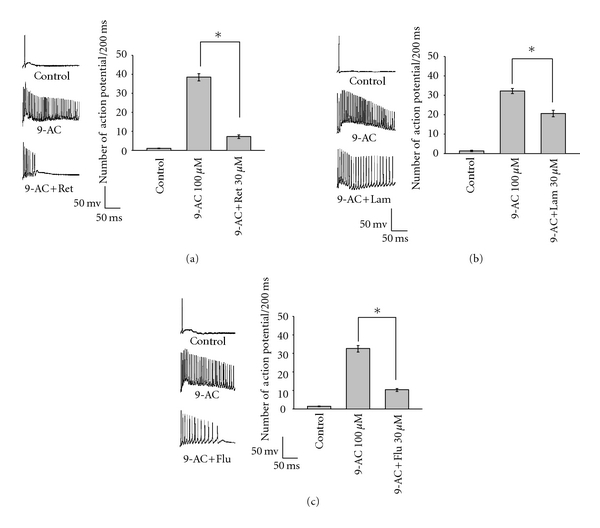
The effects of retigabine, flupirtine, and lamotrigine on the firing frequency of repetitive action potentials induced by 9-AC (0.1 mM). A train of muscle action potentials was triggered by a depolarising current injection (20 nA, 200 msec) through the recording pipette. The effects of retigabine (0.03 mM), flupirtine (0.03 mM), and lamotrigine (0.03 mM) on the firing frequency of action potentials induced by 9-AC are shown in (a, b, and c), respectively. *P < 0.01 as compared with 9-AC alone.

The mean frequencies of action potentials of sarcolemma evoked by a depolarisation square pulse stimulation (20 nA, 0.2 sec) without any chemical treatment are 1.27 ± 0.13, 1.53 ± 0.18, and 1.55 ± 0.31 Hz in the three experimental groups, respectively (Figures 6(a)–6(c): control). The application of 9-AC (0.1 mM) to skeletal muscle fibres increased the firing frequency of repetitive action potentials (38.7 ± 1.9, 32.7 ± 1.8 and 32.4 ± 1.3 Hz in the three experimental groups, resp.) (Figures 6(a)–6(c): 9-AC). Retigabine (Figure 6(a)) and Flupirtine (Figure 6(b)), but not lamotrigine (Figure 6(c)), significantly inhibit the firing frequency of action potentials induced by 9-AC (retigabine: from 38.7 ± 1.9 to 7.5 ± 0.9 Hz; flupirtine: from 32.7 ± 1.8 to 10.5 ± 0.8 Hz; lamotrigine: from 32.4 ± 1.3 to 27.0 ± 1.7 Hz).

### 3.4. Effects of KCNQ Openers on Rise of the Slope of the Action Potential

**Figure 7:**
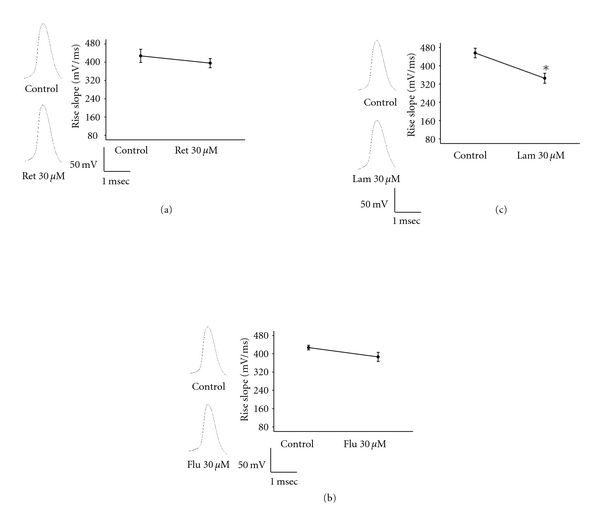
The effects of retigabine, flupirtine, and lamotrigine on the maximum rise slope of action potentials. The effects of retigabine (0.03 mM), flupirtine (0.03 mM), and lamotrigine (0.03 mM) on the maximum rise slope of action potentials are shown in (a, b, and c), respectively. *P < 0.01 as compared with before the chemical treatment.

To determine whether KCNQ4 openers affect the sodium channel, the maximum rise slopes of action potentials were measured. The results showed that retigabine and flupirtine are unable to affect the maximum rise slope of action potentials (427.1 ± 10.1 mV/ms and 386.3 ± 19.8 mV/ms, before and after treatment with retigabine, resp.; 427.6 ± 29.1 mV/ms and 395.9 ± 20.1 mV/ms, before and after treatment with flupirtine, resp.) (Figure 7). Lamotrigine slightly but significantly inhibits the maximum rise slope of action potentials (440.9 ± 21.0 and 345.4 ± 21.8 mV/ms, before and after treatment with lamotrigine, resp.) (Figure 7).

## 4. Discussion

Mutations in the CLCN1 gene cause membrane hyperexcitability in skeletal muscle. The resulting transient muscle stiffness is characterised by involuntary aftercontractions and a slowed relaxation (myotonia). This condition can also be recapitulated experimentally by blocking membrane chloride (Cl^−^) channels of normal muscle with 9-anthracene carboxylic acid (9-AC) [29, 31–33]. 9-AC causes the repetitive firing of action potentials in mammalian skeletal muscle membranes and thus induces slowed relaxation in skeletal muscle. We applied 9-AC (0.1 mM) to mouse skeletal muscle to produce an animal model of myotonia. In the present study, we compared the effects of retigabine, flupirtine, and lamotrigine on 9-AC-induced myotonia in mouse skeletal muscle. Our results showed that retigabine and flupirtine, but not lamotrigine, can significantly reduce the myotonic membrane hyperexcitability induced by 9-AC. Because retigabine and flupirtine did not cause changes in the rise slope of the action potentials, the effects of retigabine and flupirtine were not similar with sodium (Na^+^) channel blockers. It has been reported that retigabine is a selective Kv7 (KCNQ) potassium channel opener [34–36], which can act as an anticonvulsant by reducing excitability through the stabilisation of the neuronal membrane. Flupirtine also acts as a Kv7 channel opener and is a centrally acting, nonopioid analgesic with muscle relaxant properties that is advocated for use in a number of pain conditions [37]. Confocal immunoblotting analysis of the mammalian skeletal sarcolemma demonstrated the distribution of Kv 7.2 and Kv 7.3 to be in Z-line and Kv 7.4 expression to be restricted to the plasma membrane [25]. From these data, it appeared that KCNQ proteins existed on the plasma membranes of skeletal muscle. However, it is not known whether the KCNQ openers can effectively reduce the membrane overexcitability of mammalian skeletal muscle. In this experiment, we demonstrated that flupirtine, and retigabine can inhibit the membrane excitability of skeletal muscle induced by 9-AC, and these effects can be reversed by treatment with the KCNQ blocker linopirdine. Lamotrigine, a Na^+^ blocker in the central nervous system that acts without effects on KCNQ channels, has a lesser effect on the reduction of excitability induced by 9-AC. Therefore, flupirtine and retigabine inhibit membrane excitability induced by 9-AC through effects on the KCNQ channel rather than on the blockade of the Na^+^ channel. In clinical practice, various Na^+^ channel blockers have been used to treat myotonia, such as carbamazepine, mexiletine, phenytoin, and procainamide. Mexiletine is the drug of choice for reducing myotonia in myotonic dystrophy type I [38]. Na^+^ channel blockers have some unwanted effects, including a tendency to reduce muscle force by decreasing muscle action potentials. A Cl^−^ channel opener is not currently available for our use; therefore, we are utilising Na^+^ channel blockers to treat myotonia. Although lamotrigine has been shown to act at voltage-sensitive Na^+^ channels and stabilise neural membranes, it has a lesser effect on muscle myotonia as compared with KCNQ openers (e.g., retigabine and flupirtine). The effect of lamotrigine may be more selective in the central nervous system than in peripheral tissues. Evidence from this study suggests that KCNQ openers may be a new class of drugs for the treatment of myotonia. Because retigabine and flupirtine cannot significantly change the rise slope of action potentials, the unwanted effects on Na^+^ channels can thus be excluded. All of these studies indicate that retigabine and flupirtine may be considered useful in the treatment of myotonia.
